# Active Cellulose-Based Food Packaging and Its Use on Foodstuff

**DOI:** 10.3390/polym16030389

**Published:** 2024-01-31

**Authors:** Anamaria Irimia, Vasile Cristian Grigoraș, Carmen-Mihaela Popescu

**Affiliations:** 1 Petru Poni Institute of Macromolecular Chemistry of the Romanian Academy, Grigore Ghica Voda Alley, 41A, 700487 Iasi, Romania; crgrig@icmpp.ro; 2 Wood Science and Technology, School of Computing, Engineering and the Built Environment, Edinburgh Napier University, Unit 1, Seven Hills Business Park, 37 Bankhead Crossway South, Sighthill, Edinburgh EH11 4EP, UK

**Keywords:** cellulose-based Kraft paper, active food packaging, plant oils, radiation-induced surface activation, food safety/testing

## Abstract

The essential role of active packaging is food quality improvement, which results in an extension of shelf life. Active packaging can also further enhance distribution from the origin point, and contributes to food waste reduction, offering greater sustainability. In this study, we introduced a new method for obtaining cellulose-based active packages, combining gamma irradiation as an eco-friendly activation process, and clove essential oil and cold-pressed rosehip seed oil as bioactive agents. Newly obtained bioactive materials were evaluated to assess their structural, hydrophobic, and morphological properties, thermal stability, and antioxidant and antimicrobial properties. The results showed that the plant oils induced their antimicrobial effects on paper, using both in vitro tests, against several bacterial strains (Gram-positive bacteria *Listeria monocytogenes* and Gram-negative bacteria *Salmonella enteritidis* and *Escherichia coli*), and in vivo tests, on fresh cheese curd and beef. Moreover, these oils can help control foodborne pathogens, which leads to extended shelf life.

## 1. Introduction

The presence on the market of new food products, and the need for constant verification of their quality until consumption, along with the need for reducing food contamination during storage time, have led to the recent development of different packaging technologies, such as active packaging. Active packaging is defined as “packaging in which subsidiary constituents have been deliberately included in or on either the packaging material or the package headspace to enhance the performance of the package system” [1]. Oxygen scavengers, flavor-releasing/absorbing systems, antioxidants, antimicrobials, and moisture controllers are of great importance for the active packaging systems. These systems change the environmental conditions of food during the preservation period. Their advantages for food applications include an extended shelf life, which translates into a later expiration date and a reduction in food waste, maintaining the quality of packaged foods at the same time [2].

Plastics are the most common materials used in active food packaging applications, mainly for products with a short shelf life [3]. However, most of the plastic products are single-use with low service life, and due to their long-lasting stability in environmental conditions and thermal stress, they present difficulty in disposal and recycling. On the other hand, cellulose is an environmentally friendly material which can be used for food packages. Nonetheless, native cellulose is hardly used in food packaging applications due to its strong hydrophilicity, pure solubility, and high crystallinity. Hence, cellulose blends/composites with other polymers are preferred in this area of application. Generally, cellulose-based packaging materials are largely used for wrapping, presenting the advantage of natural sources, easy biodegradability, and recyclability [4,5,6,7,8].

To achieve active food packaging, a variety of natural additives, such as antimicrobial, antioxidant, coloring, or sweetener agents, can be used [9,10].

Plant-extracted oils present high interest due to their biological activities and their possibility for replacing different chemical preservatives in food. Essential oils represent a complex mixture of compounds, where the main components include alkaloids, flavonoids, isoflavones, monoterpenes, phenolic acids, carotenoids, and aldehydes, with well-known antioxidant and antimicrobial activity [11,12]. However, they do not have photothermic resistance and are more likely to present oxidative decomposition, thus limiting their application. To reduce the volatility, but at the same time maintain biological activity, essential oils can be immobilized on different surfaces that are suitable for food packaging applications [4,8]. Clove essential oil proved its efficiency as an antimicrobial agent against a large number of microorganisms like Gram-positive bacteria (e.g., *Staphylococcus aureus*, *Streptococcus*, *Listeria monocytogenes*) or Gram-negative bacteria (e.g., *Escherichia coli*, *Salmonella*, *Pseudomonas aeruginosa*) and fungi (e.g., *Aspergillus*, *Penicillium*) [13,14].

Plant oils extracted from seeds present lipophilic character induced by the presence of saturated and unsaturated fatty acids, but also antioxidant and antimicrobial activity induced by other compounds (such as phenolic derivatives and flavonoids). Cold-pressed rosehip seed oil (*Rosa canina* L.) consists of more than 90% of unsaturated fatty acids in its composition, but also presents some phenolic derivatives, tocopherols, and carotenoids. The oil’s composition induces its bioactive properties, such as good antioxidant properties and high antimicrobial activity [15,16].

The mechanism of action of gamma (ionizing) irradiation on polymeric-based materials implies the formation of radicals. These may interact with each other, or with atmospheric gases, as well as with other compounds, and may lead to different processes such as polymerization, crosslinking, grafting, or degradation [17,18]. The type of modification which may occur in the polymeric-based material can be influenced by several factors, including the polymeric chemical structure, irradiation dose, or surrounding environment [19,20]. Generally, for the sterilization and preservation of food, low doses of gamma irradiation (from 2.5 up to 25 kilogray) are applied [21,22].

In many cases, gamma irradiation is chosen as an ideal physical treatment for the surface modification of polymers because, aside from being environmentally friendly, under optimal mild conditions it selectively modifies the surface layers of the treated materials [23,24,25].

In our previous works, gamma irradiation was successfully used to functionalize efficiently different polymeric-based substrates (such as cellulose/chitin mixed fibers, unbleached Kraft paper, and polylactic acid) to enable the immobilization of several bioactive agents (e.g., phenolic compounds, essential, or cold-pressed oils) [5,9].

This study aimed to obtain new active packages from eco-friendly and biodegradable materials with antioxidant and antibacterial properties. The results showed that the studied materials are able to control the foodborne pathogens in specific edibles, leading also to their extended shelf life.

## 2. Materials and Methods

### 2.1. Materials

Commercial bleached Kraft paper (BKP) with a density of 0.6 g/cm^3^ and a thickness of 100 µm was purchased from Adi Center SRL, Iasi, Romania.

N-hydroxysuccinimide (NHS) (used as coupling agent) and 1-ethyl-3-(3-dimethylaminopropyl) carbodiimide hydrochloride (EDC) (used as an activation agent) were purchased from Sigma-Aldrich (Steinheim, Germany).

Analytical grade methanol and chloroform were used as solvents and were purchased from Chemical Company, Iasi, Romania.

Clove essential oil (CO) was purchased from Fares, Orastie, Romania, and cold-pressed rosehip seed oil (RO) was purchased from S.C. Herbavit S.R.L., Iasi, Romania. These two oils were chosen for the functionalization of the bleached Kraft paper. Their selection was made based on their high content on antioxidant compounds, as observed in our previous work [12].

The main compounds in clove essential oil are eugenol (about 86%), eugenol acetate (about 8%), and β-caryophyllene (about 4.5%) [26]. Meanwhile, rosehip seed vegetal oil is mainly composed of linoleic acid (54%), α-linolenic acid (about 22%), and oleic acid (about 19%), which are unsaturated fatty acids [27].

### 2.2. Functionalization of the Bleached Kraft Paper

Commercial bleached Kraft paper activation was achieved using γ-irradiation. The process took place in air at room temperature, inside of a γ-irradiator M-38 GAMMATOR (Best Theratronics, Ottawa, ON, Canada), equipped with a ^137^Cs source. The dose rate was 0.4 kilogray/h (kGy/h), achieving doses of γ-irradiation of the material between 5 and 20 kGy. To achieve homogenous irradiation and to convert the γ-rays into γ-electrons for a more efficient energetic transfer, the samples were continuously rotated and covered with aluminum foil.

The active centers formed on the material surface after γ-irradiation present high reactivity and can easily form new bonds [4,25]. During air exposure they are transformed into reactive functional groups that are further able to interact via chemical or physical bonds with different bioactive compounds.

**Scheme 1:**
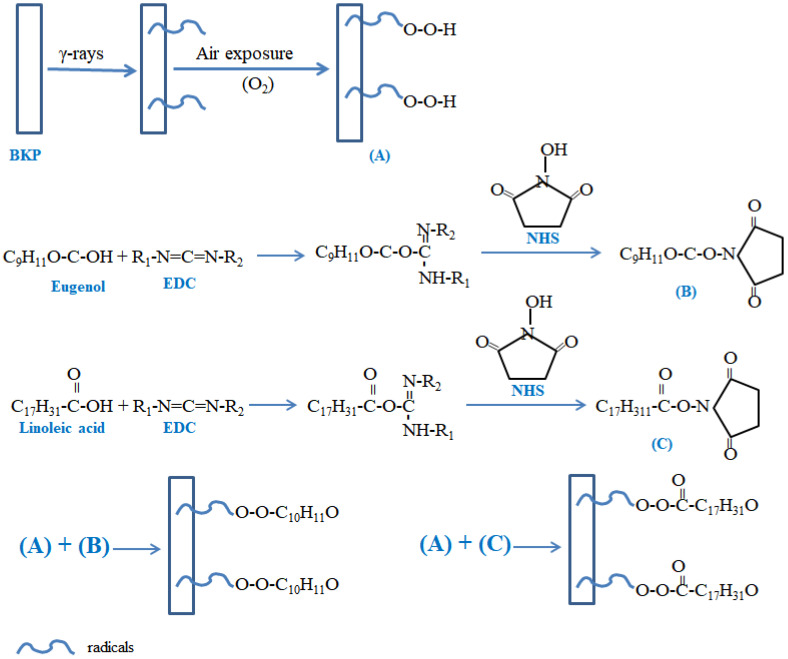
Surface modification of BKP with vegetable oils using gamma irradiation.

After activation, the paper was immersed into chloroform solution (10 wt%) containing cold-pressed rosehip seed oil or into methanol solution (10 wt%) containing clove essential oil, under mechanic stirring for 60 min. To achieve grafting, the plant oil solutions were activated using two coupling agents, namely 1-(3-dimethylaminopropyl)-3-ethylcarbodiimide (EDC) and N -hydroxysuccinimide (NHS). As a carboxyl group activator (such groups belong to the structure of some components of plant oils) for the coupling of primary amines to form amide bonds, EDC was used. Further, in order to increase the coupling efficiency and form stable amine reactive products, NHS was used (Scheme 1).

The modified paper was dried in an oven at 60 °C, and then washed with chloroform or methanol to eliminate unreacted chemicals that were only physically adsorbed. Finally, the materials were dried in a vacuum oven at 40 °C and analyzed.

### 2.3. Investigation Methods

#### 2.3.1. ATR-FTIR Spectra

ATR-FTIR spectra were recorded at 4 cm^−1^ resolution and 64 scans, in the 4000–600 cm^−1^ wavenumber range, using a Bruker VERTEX 70 spectrometer (Bruker Optics, Ettlingen, Germany) equipped with a ZnSe crystal. For each sample, an average spectrum obtained from three recorded spectra was used for the evaluation.

#### 2.3.2. SEM—EDAX Analysis

SEM—EDAX analysis was achieved using a scanning electron microscope SEM/ESEM—EDAX QUANTA 200 (FEI Company, Portland, OR, USA), at 1000× magnification and no further treatments of the samples. EDAX results were based on the average of three measurements taken in different sample areas.

#### 2.3.3. Water Contact Angle Measurements

Water contact angle (WCA) measurements were used to highlight the influence of the plant oils on the hydrophobicity of surface paper. A surface is considered hydrophobic if the water contact angle is larger than 90°, and hydrophilic if the water contact angles are smaller than 90°. For practical applications in the food industry, it is important to evaluate the WCA of the samples surface [28]. In this study, the surfaces’ wettability was evaluated by static contact angle measurements performed on a CAM-200 goniometer (KSV Instruments Ltd., Helsinki, Finland). The WCA was determined by the sessile drop method at room temperature and controlled humidity within 5 s after placing 1 μL drops of liquid on the samples’ surface. For each sample, the results from a minimum of 10 measurements and three different replicates were considered for the statistical determination of the final average value.

#### 2.3.4. Differential Scanning Calorimetry (DSC)

The glass transition temperature (Tg) and the melting temperature (Tm) of the samples were determined with a differential scanning calorimeter (Mettler DSC 30, Mettler-Toledo, Greifensee, Switzerland). The apparatus was calibrated with pure indium, lead, and zinc at various scanning rates. Heating and cooling scans were performed at a 10 °C/min heating rate on ~4 mg of sample in nitrogen atmosphere (40 mL/min).

#### 2.3.5. DPPH Radical Scavenging Assay

The radical scavenging activity of the paper was measured using the DPPH (2,2-diphenyl-1-picrylhydrazyl), which is a stable violet color free radical, which turns yellow under the action of proton donating compounds. The color change can be monitored at 517 nm using a UV spectrometer. The radical scavenging activity was calculated using the same equation as in our previous paper [29].

#### 2.3.6. In Vitro Antibacterial Activity

Three different American Type Culture Collection (ATCC, Rockville, MD, USA) bacterial strains were used for antibacterial tests according to specific standard methods. These were *Escherichia coli*—ATCC 25922 (Gram −), *Salmonella enteritidis*—ATCC 14028 (Gram −), and *Listeria monocytogenes*—ATCC 7644 (Gram +). Briefly, the procedure involved (i) sterilization of the samples in an autoclave at 110 °C, 0.5 bars and for 20 min, (ii) contamination of their surface by seeding 0.1 mL bacterial cultures, (iii) inoculation and incubation at 37 °C for two different periods of 24 and 48 h, followed by (iv) colony counting. For the identification of *E. coli* we used coloration with 5-bromo-4-chloro-3-indolyl-β-D-glucuronide; for *L. monocytogenes* we used the β-hemolysis test, while for the identification of *S. enteritidis* we used the counting of the specific plate count Xylose Lysine Deoxycholate agar (XLD agar) method.

#### 2.3.7. Microbiological Assessment on Food

We aimed to test packaging materials with antimicrobial properties on a traditional dairy product (fresh curd from an authorized producer) and fresh beef (originating from one local slaughterhouse delivered within 4 h after slaughter) in aseptic laboratory conditions. The motivation for choosing these foodstuffs consists of the particularities related to the constant production, the form of presentation, the short-term validity, and the increased adaptability to the proposed techniques.

Foodstuffs were tested with appropriate sampling corresponding to the physico-chemical and microbiological examinations following the legislation in force, and were characterized by a pleasant smell with a specific aroma, no foreign smell; pH = 5.1 for curd and pH = 5.7 for beef, which pointed out their good hygienic standards during production, as well as their quality.

The bioactive paper packages cut into pieces of 4 × 4 cm size were placed in sterile Petri dishes. Next, food samples aseptically cut into 1 × 1 × 1 cm pieces were placed in the center of the paper. After that, the Petri dishes were sealed and stored in a refrigerator at 7 °C. After 24 and 48 h, respectively, the samples were taken out from the refrigerator to room temperature and further analyzed. The pH of the food samples’ surface which was in contact with the packaging was checked using indicator paper. On the other side, the surface of the active packaging which was in contact with the food samples was wiped off with a sterile swab and then immersed in a test tube with 10 mL of physiological serum. Volumes of 1 mL from the formed suspensions were seeded in two Petri dishes containing plate count agar (PCA). After solidification, the plates were thermostated at 30 °C for 72 h according to the SR ISO 4833/2014 standard [30]. The SR EN ISO 7218/2014 standard [31] was used for the microbiological examinations and interpretation of the results.

## 3. Results and Discussion

### 3.1. ATR-FTIR Spectra Results

**Figure 1:**
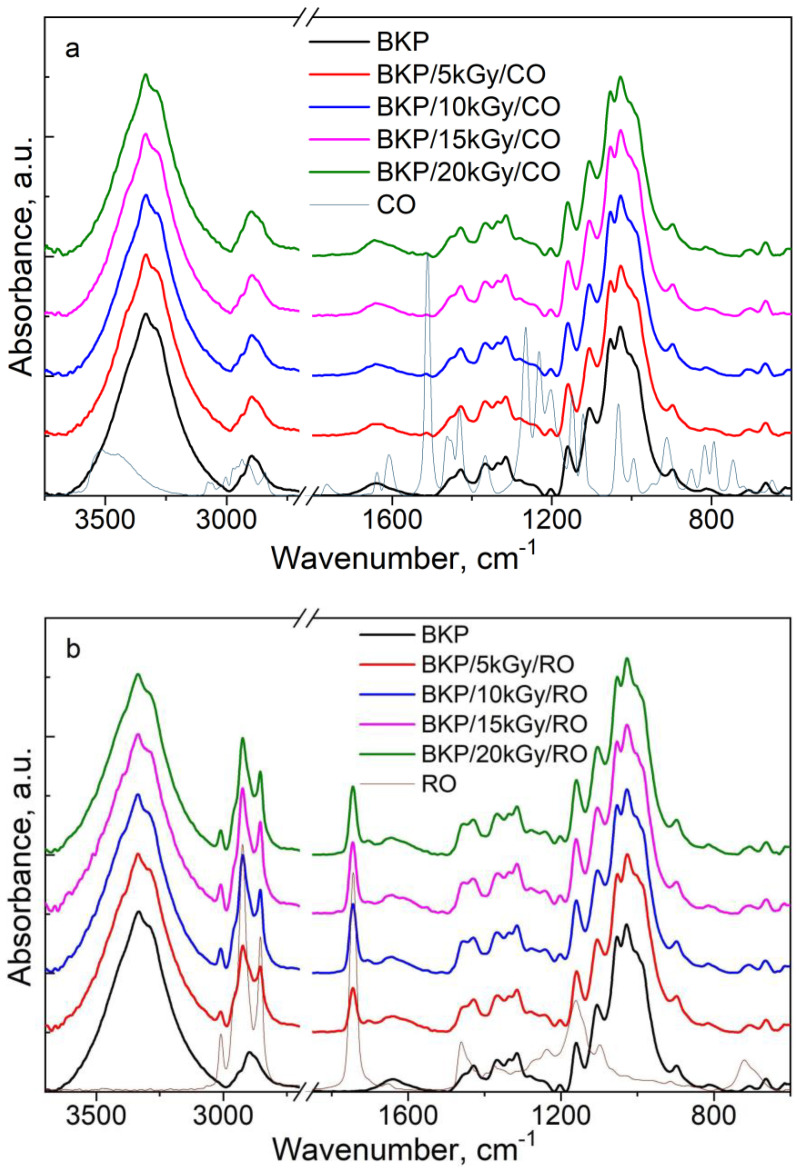
ATR-FTIR spectra of bleached Kraft paper modified with (**a**) clove essential oil (BKP/CO) and (**b**) cold-pressed rosehip seed oil (BKP/RO) by using gamma irradiation.

The ATR-FTIR spectra of the bioactive Kraft paper were recorded and are presented in Figure 1. They present two main regions: 3800–2700 cm^−1^ assigned to different OH stretching vibrations as well as to CH_2_ and CH_3_ symmetric and asymmetric stretching vibrations and the 1800–600 cm^−1^ region assigned to different stretching and deformation vibrations of different groups belonging to cellulose (from paper) and the used oils.

The spectrum of BKP is a typical spectrum of cellulose, with the first region (between 3700 and 2700 cm^−1^) assigned to different inter- and intramolecular stretching vibrations of hydrogen bonds from cellulose structure, as well as to methyl and methylene groups. The second region presents the main bands at: around 1640 cm^−1^ (assigned to OH deformation vibration of adsorbed water), 1430 cm^−1^ with a shoulder at 1452 cm^−1^ (assigned to C–H deformation vibration), 1365, 1001, and 895 cm^−1^ (assigned to CH deformation and CH_3_ symmetric deformation), 1336 and 1316 cm^−1^ (assigned to CH_2_ rocking vibration), 1204, 1105, and 986 cm^−1^ (assigned to C–O stretching vibration), 1161 cm^−1^ (assigned to the C–O–C stretching vibration mode of the pyranose ring), 1053 cm^−1^ (assigned to C–O stretching mainly from C(3)–O(3)H), and 1029 cm^−1^ (assigned to C–O and C–C stretching ring vibration) [32,33].

On the other hand, the spectrum of the clove oil (thin line in Figure 1a) presents its characteristic bands as follows: a large band with two maxima at 3514 and 3448 cm^−1^ (assigned to hydroxyl groups’ stretching vibrations), several bands between 3100 and 2800 cm^−1^ (assigned to CH stretching vibrations), and bands at 1638 cm^−1^ (assigned to C=C stretching vibration of vinyl groups), 1610 and 1512 cm^−1^ (assigned to C=C stretching vibration in an aromatic ring), 1457 and 1430 cm^−1^ (assigned to CH deformation vibration), 1367 cm^−1^ (assigned to C-H deformation vibration in eugenol methyl), 1267 cm^−1^ (assigned to C–O group stretching vibration of phenolic hydroxyl), 1202, 1148, and 1122 cm^−1^ (assigned to C–O stretching vibrations of ether and alcohol functional groups), 1232 cm^−1^ (assigned to stretching vibration of C–C groups), 1033 and 995 cm^−1^ (assigned to C–O–C groups’ stretching vibration of aromatic ether), 913 cm^−1^ (assigned to O-H bending vibrations), and 851, 817, and 793 cm^−1^ (assigned to bending vibration of the C-H groups from the aromatic ring). The main component of the clove oil is represented by eugenol, a molecule which contains phenolic hydroxyl groups, aromatic rings, double bonds, and ketone and carboxyl groups [17,19,34].

When the paper was modified with clove essential oil BKP/CO (see colored contours in Figure 1a) some differences were observed, as follows: new bands or shoulders with maxima at around 2940–2945 cm^−1^ (assigned to the stretching vibration of the CH groups in cellulose, but also to groups belonging to aromatic structures from the oil), at 1516 cm^−1^ (assigned to stretching vibrations of the C=C from the aromatic ring), a shoulder at around 1266 cm^−1^ (assigned to –C–O–C and C–O deformation vibrations from cellulose and groups from the oil), a band at around 811–813 cm^−1^ (assigned to CH stretching vibrations). In addition, an increase of the intensities of the bands from 1640 cm^−1^ (assigned to OH deformation vibrations from adsorbed water molecules (in cellulose), but also to –COOH and C=O stretching vibrations from CO oil), 1452 cm^−1^ (assigned to –CH deformation vibrations), 1241 cm^−1^ (assigned to OH deformation vibrations), and 1160 and 1051 cm^−1^ (assigned to –C–O–C and C–O groups stretching vibrations), were observed. These modifications are due to the overlapping of the bands belonging to the cellulose as well as to CO oil.

RO spectrum, presented as a thin line in Figure 1b, shows bands at 3009, 2926, and 2855 cm^−1^ (assigned to –CH_2_ asymmetric and symmetric stretching vibrations), at 1743 cm^−1^ (assigned to stretching vibration of carbonyl (C=O) from esters), 1461 cm^−1^ (assigned to CH_2_ deformation vibrations), 1237, 1161, and 1098 cm^−1^ (assigned to C–O stretching vibrations), and at 722 cm^−1^ (assigned to CH_2_ groups in plane deformation vibrations) [21].

In the case of RO modified paper, new bands or shoulders with maxima at 3011 and 2851 cm^−1^ (assigned to CH and CH_2_ stretching vibrations), and at 1744 and 1705 cm^−1^ (assigned to different –COOH and C=O groups stretching vibrations, but also to C=O from amide I groups), were observed. Aside from these new bands, there was an increase in the intensity of the bands from 2921 and 2855 cm^−1^ (assigned to CH_2_ and CH_3_ group stretching vibrations), 1455 cm^−1^ (assigned to CH_2_ deformation vibrations), 1242 cm^−1^ (assigned to C–O stretching vibrations from ester groups), 1159 and 1105 cm^−1^ (assigned to C–O–C and CO stretching vibrations), and 715 cm^−1^ (assigned to CH_2_ groups in plane deformation vibrations), that was observed in the spectra of the treated paper with RO oil.

The appearance of the band from 1705 cm^−1^ can indicate the conversion of amine groups (appearing on the surface of the Kraft paper after γ-irradiation in air) into amide groups after the reaction with RO oil, which was facilitated by the coupling agents.

All these modifications observed in the spectra of the treated papers indicated the structural changes appearing after activation by gamma irradiation with both oils, the efficiency being higher when a 20 kGy irradiation dose was used. Moreover, the variations of the surface modification are related to the composition of the used plant oils.

### 3.2. SEM Results

**Figure 2:**
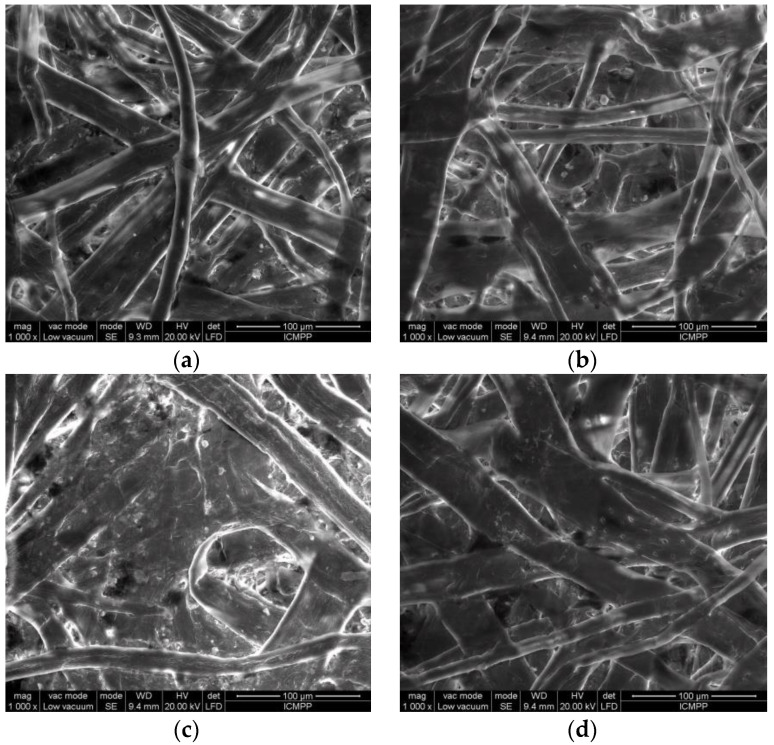
SEM images of the analyzed samples: (**a**) BKP, (**b**) BKP/20 kGy, (**c**) BKP/20 kGy/CO and (**d**) BKP/20 kGy/RO.

The morphology of the surface of modified BKP by using γ-irradiation activation was analyzed by SEM—Figure 2.

SEM images of untreated bleached Kraft paper presented a dense fiber matrix, with cellulose fibers tied together and closely packed. In the case of the activated paper, the surface of the fibers appeared smoother compared to unactivated paper. Further, the modified BKP shows that the vegetable oils entered the pores, leading to an increase in homogeneity. Hence, liquid penetration (such as water) through modified Kraft paper was expected to be reduced.

The surface composition elemental analysis gives information concerning the modification of the paper. Table 1 summarizes the modified samples’ EDAX results compared to unmodified ones.

As expected, the main elements in the sample composition were carbon and oxygen.

On the surface of the unmodified samples, a negligible amount of nitrogen was detectable by EDAX. Instead, nitrogen was present in significant amounts on the surfaces of the modified samples, representing proof of Kraft cellulose paper modification.

Pre-treatment using γ-irradiation induced a high decrease in the content of carbon on the paper surface. Additionally, the recorded content of the oxygen was higher, showing an increased O/C atomic ratio. The content of the nitrogen was slightly higher after using γ-irradiation. This may indicate that the nitrogen from the air was also involved in the formation of reactive centers on the paper surface.

The overall O/C ratio was increased after γ-irradiation and modification with plant oils. The increase was more obvious after the addition of rosehip oil when compared to the untreated paper. This may indicate an increased polarity due to the presence in the oils’ composition of the oxygen-containing groups.

### 3.3. Water Contact Angle Measurements

**Figure 3:**
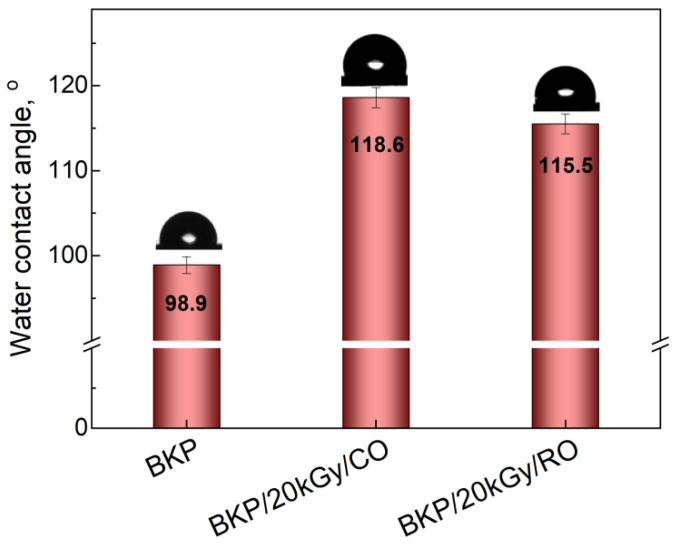
Water contact angles and images of static water contact angles of bleached Kraft paper treated with plant oils compared to untreated paper.

Figure 3 shows the WCA values and the photographs of water droplets deposited onto studied surfaces for unmodified and plant oil-modified bleached Kraft paper.

The water contact angle of unmodified bleached Kraft paper was of 98.9°, indicating a slight degree of hydrophobicity (see Figure 3) of its surface. The water contact angle values were significantly increased after modification (over 116°), especially for clove oil-modified paper. This indicates that coated paper has high hydrophobicity, which can be explained by the hydrophobic nature of the oils used in this study.

The water contact angle value for the BKP/CO is higher compared to the BKP/RO one. Eugenol (C_10_H_12_O_2_), the main active compound in clove oil, is a molecule with a smaller molecular chain than linoleic acid (C_18_H_32_O_2_), the main component from rosehip seed oil. Thus, it can create more intermolecular bonds with the paper substrate, leading to an increase of hydrophobic functional groups on the papers’ surface. As a result, the interactions between clove oil and Kraft paper substrate are stronger compared with those between rosehip seed oil and paper substrate.

### 3.4. DSC Results

**Figure 4:**
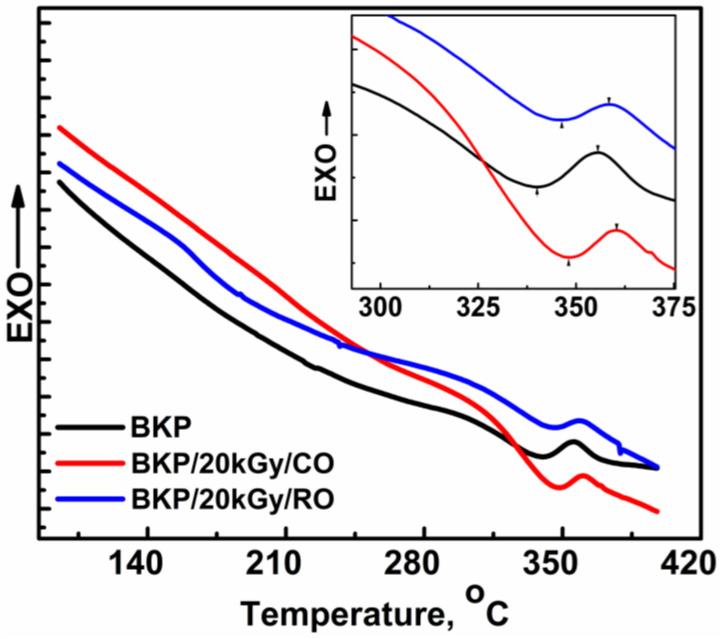
DSC curves for the analyzed samples between 80–400 °C and magnification from 260 to 400 °C.

The thermal behavior of BKP, BKP/CO, and BKP/RO using DSC analysis is shown in Figure 4. The curves show two processes, firstly an endothermic one, followed directly by an exothermic one.

The cleavage of the glycosidic bonds (which ensures the stability of cellulose structure) is assigned to the destruction of the cellulose crystalline network, where the melting process occurs. It is worth mentioning that bleached cellulose contains a more compact crystalline structure after removing most of the amorphous fraction (lignin content) using a pretreatment process [22]. Thus, cellulose crystals undergo a reorientation process and finally melt, as shown by the endothermic process in the DSC signal. This process has been accompanied by glycosidic bonds breaking simultaneously with the formation and instant volatilization of levoglucosan molecules. Such thermal behavior (observed from the DSC curves as an exothermic process) is specific to depolymerization, and it means the thermal decomposition of cellulose [35].

The irradiation doses slowly increase the thermal stability of paper, as the data in Table 2 show. The chemical reaction between resulted active radicals and vegetable oil molecules results in the papers’ stabilization against their thermal degradation.

Clove essential oil, being more volatile, becomes more stable at higher temperatures if it is attached to a surface having higher thermal stability [36,37]. The higher thermal stability of rosehip seed oil (up to 340 °C) has been proved by other authors and explained by its high content of tocopherols; results show that degradation starts after this temperature [16]. DSC results for BKP/CO showed an increase in the maximum endothermic process from 340 °C to 348.1 °C (Table 2). Similar thermal behavior was reported by Irimia et al. [38], where a cellulose-based material treated with eugenol showed the same thermal stability, obtained from TG/DTG measurements. The modification of BKP with clove oil affects the delay of the cellulose cleavage of glycosidic bonds (melting). Analyzing DSC curves, the same effect is observed on BKP/RO, but here the increase of the maximum endothermic peak value was slightly reduced (346.4 °C)—Table 2. An explanation could be provided by the intermolecular bonding between the cellulose fibers’ surface of BKP and eugenol molecules (contained by CO) or linoleic acids as the major constituent of RO. As a result, these molecules will induce an obstruction effect which consumes additional thermal energy, thus delaying the temperature increase on cellulose fibers during the temperature scan. This delay, which occurs due to a slightly higher thermal diffusivity, affects both the endothermic and the exothermic processes.

There is a small difference that is not negligible, which can be assigned to the difference between the chemical structures of eugenol and linoleic acid molecules. The smaller volume of the eugenol molecule favored its easier penetration into the spaces between the cellulosic fibers on the BKP surface. This led to the establishment of a greater number of bonds between clove oil and BKP, ultimately consuming a higher amount of thermal energy than simple BKP. On the other hand, the linoleic acid molecules, whose higher volume imposed by its linear structure can establish fewer bonds, had a slightly lower effect on the processes observed in the DSC curves.

Finally, based on these statements established after the analysis of the results from DSC scans, we can conclude that clove oil molecules prevent more temperature increase than rosehip seed oil.

### 3.5. DPPH Radical Scavenging Assay of Analyzed Samples

The DPPH tests were performed in order to evaluate whether the plant oils impart their antioxidant activity to bleached Kraft paper after modification (Table 3).

The antioxidant activity is strongly dependent on the efficiency of the individual plant oil to modify the paper substrate and give a stable non-radical product. It is believed that the hydroxyl groups, which are present in the structure of the oils, induce their antioxidant activity.

Kraft paper substrate modified with clove oil (BKP/CO) presented higher antioxidant activity, as indicated by the low value for IC50 concentration. This behavior might be due to the high content of eugenol and eugenol acetate phenolic compounds from its structure, which make the main contribution to its total antioxidant activity [39].

The main radical scavenger compounds from rosehip seed oil responsible for the antioxidant activity are phenols, flavonoids, phenolic acids, anthocyanins, and tannins [40,41], which represent less than 5 wt% from its composition, the rest (over 95 wt%) being fatty acids [39]. This is why BKP/RO showed a relatively low antioxidant activity compared to BKP/CO.

Similar results were obtained in our previous studies, where cellulose-based materials were modified with different phenolic compounds using cold plasma or gamma irradiation for substrate activation [4,8].

### 3.6. In Vitro Antibacterial Activity

Gamma irradiation greatly influences antibacterial activity. It is known as a relatively simple and safe microbial sterilization method used in various applications for being non-polluting and having low operating costs.

**Figure 5:**
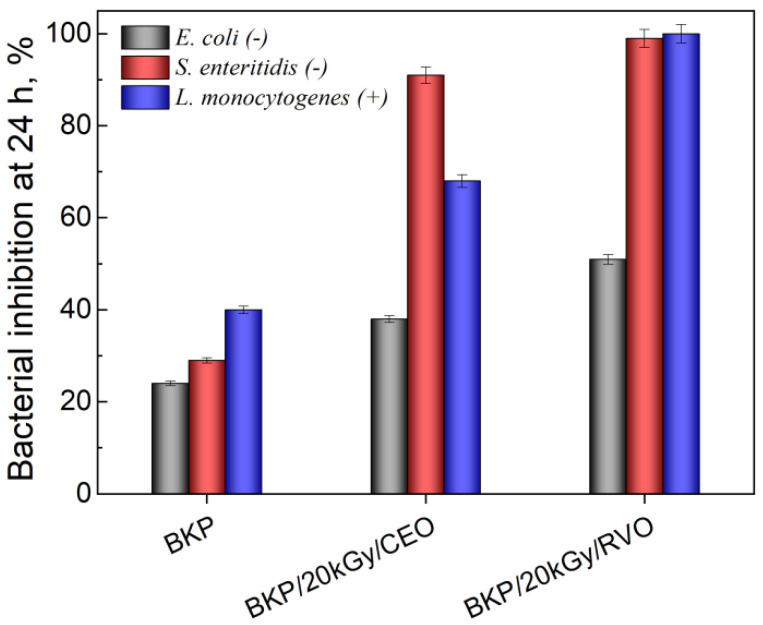
Bacterial inhibition percentage after 24 h determined for bleached Kraft paper modified by plant oils compared to untreated paper.

The antimicrobial test results for the inhibition of *Escherichia coli* (Gram −), *Salmonella enteritidis* (Gram −), and *Listeria monocytogenes* (Gram +) bacteria are presented in Figure 5.

From Figure 5, it can be concluded that modification of BKP by plant oils using γ-irradiation significantly increased the inhibition at 24 h for tested bacteria. The antimicrobial activity reached up to 100%.

Differences were observed between the two used plant oils. RO showed higher bacterial inhibition when compared with CO for all tested bacteria, probably due to the presence of antibacterial phenolic and flavonoid compounds in its structure. Generally, bioactive compounds were very successful in exerting antimicrobial effects against *S. enteritidis*. The efficiency increased in the *E. coli* < *L. monocytogenes* < *S. enteritidis* order.

### 3.7. Microbiological Evaluation on Fresh Food

Our aim was the comparative analysis of modified packaging materials’ behavior against the usual ones, on the same food, in environmental conditions specific to ordinary marketing, through appropriate microbiological examinations, following the changes that occurred at 24 and 48 h after contact with those packages.

Packaging materials with proven antimicrobial properties were tested in aseptic laboratory conditions on a traditional dairy product, namely fresh curd, and on fresh beef. The reason for choosing these types of products consists of the particularities related to their constant production, the form of presentation, and their short shelf life.

**Figure 6:**
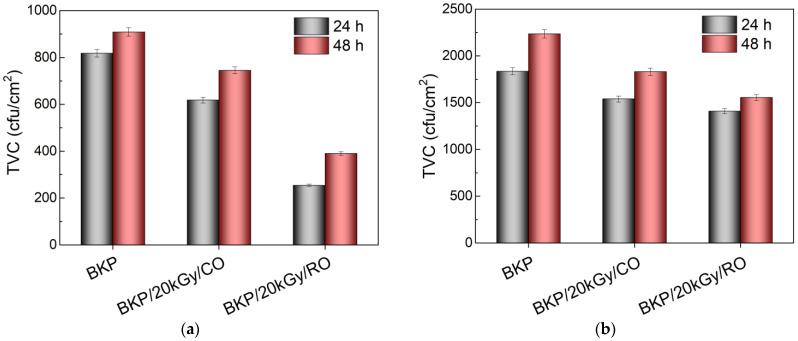
Total Viable Count of unmodified BKP and BKP/CO and BKP/RO for (**a**) fresh curd and (**b**) fresh beef.

Figure 6 presents the population dynamics of cheese and meat spoilage-related microorganisms, after 24 and 48 h, in terms of Total Viable Count (TVC).

BKP/CO and BKP/RO strongly decreased the microbial growth, as exhibited by the decrease in the total viable count of fresh curd and beef.

Both CO and RO proved to be valuable antimicrobial agents for delaying the spoilage of white cheese and beef. However, differences could be noticed between the two plant oils, with RO having a slightly better effect than CO.

Moreover, both BKP/CO and BKP/RO showed better microbial inhibition for fresh curd, where the cellular growth decreased below 45% after 48 h. However, the cellular growth remained high for fresh beef at about 70% after 48 h. These results indicate that the used plant oils to modify the bleached Kraft paper are more applicable in prolonging the shelf life of food products with relatively low bacterial content, like fresh cheese. Similar results were obtained in our previous study [8].

## 4. Conclusions

Active packaging is a promising direction from the food packaging development perspective. Antimicrobial agents are introduced into packaging by coating the surface of the packaging material with active compounds such as antimicrobials, antioxidants, or desiccants, or by their direct incorporation within the packaging film.

This study presents the achievement of new active food packaging using gamma irradiation as an eco-friendly substrate activation technique. Structural, morphological, and thermal properties have been evidenced by ATR-FTIR, SEM-EDAX, and DSC data.

Newly obtained bioactive cellulose-based packaging presented different antimicrobial and antioxidant properties depending on the plant oil type used. Essential clove oil induced higher antioxidant activity, while cold-pressed rosehip seed oil was more effective for in vitro (against *Listeria monocytogenes* (Gram +), *Salmonella enteritidis* (Gram −), and *Escherichia coli* (Gram −)) and in vivo antimicrobial (on fresh curd and beef) tests.

These results proved that these materials can be used in the food industry as active packages.

## Figures and Tables

**Table 1:** EDAX data for bleached Kraft paper treated with vegetable oils in comparison with unmodified paper.

Sample	C		O		N		O/C
	Wt%	At%	Wt%	At%	Wt%	At%	At%
BKP	57.12	65.51	41.78	33.72	0.50	0.48	0.51
BKP/20 kGy	54.17	61.20	44.19	37.63	0.75	0.73	0.61
BKP/20 kGy/CO	54.60	62.20	42.08	35.53	1.99	1.76	0.57
BKP/20 kGy/RO	55.39	63.75	41.83	34.38	2.42	1.48	0.54

Wt% = weight percent; At% = atomic percentage.

**Table 2:** DSC parameters obtained for bleached Kraft paper treated with plant oils compared to untreated paper.

T_max_ (°C)	BKP	BKP/20 kGy/CO	BKP/20 kGy/RO
Endotherm	340	348.1	346.4
Exotherm	355.6	360.4	358.4

**Table 3:** The values of sample concentration required to scavenge 50% of DPPH free radicals (IC50).

Sample	IC50, mg/mL
BKP	-
BKP/20 kGy/CO	0.205
BKP/20 kGy/RO	95.289

## Data Availability

Data are contained within the article.
